# Phenotypic changes of interstitial cells of Cajal after intestinal obstruction in rat model

**DOI:** 10.1590/1414-431X20198343

**Published:** 2019-10-14

**Authors:** Lin Li, Changlin Zou, Zhenli Zhou, Ximo Wang, Xiangyang Yu

**Affiliations:** 1 Department of Gastrointestinal Surgery, Tianjin Nankai Hospital, Tianjin, China; 2 Tianjin Medical University NanKai Hospital, Tianjin, China

**Keywords:** Intestinal obstruction, Interstitial cells of Cajal, Stem cell factor, c-Kit protein

## Abstract

The objective was to study the effect of mechanical intestinal obstruction in rats on the phenotype of interstitial cells of Cajal (ICC). Healthy Wistar rats were randomly divided into sham-operation group (C), one day obstruction group (M1), two days obstruction group (M2), and three days obstruction group (M3), with 10 rats in each group. The expression of SCF mRNA and c-Kit protein in intestinal tissue was investigated by RT-PCR and immunohistochemistry. Compared with the sham-operation group, the relative expression of SCF mRNA and the expression of c-Kit protein in intestinal tissue were significantly decreased in both obstruction groups. Levels decreased gradually with the prolongation of obstruction time, and significantly decreased on the 3rd day after obstruction (P<0.05). Immunohistochemical staining of the small intestine showed that the number of ICC in the sham-operation group was the highest, and they were gradually decreased with the extension of obstruction time in the M1 to M3 groups. There was a significant difference between groups (P<0.05). Intestinal obstruction caused a decrease in the concentrations of SCF mRNA and c-Kit protein in ICC. With the prolongation of intestinal obstruction, the number of ICCs gradually decreased.

## Introduction

Intestinal obstruction (IO) is an important cause of intestinal motility disorders. Etiologies include adhesions (65%), hernias (10%), neoplasms (5%), Crohn's disease (5%), and others (15%). Intestinal dilatation mainly occurs in the proximal end of obstruction, increasing mural tension, reducing mucosal perfusion, causing bacterial proliferation, and reducing mural tensile strength that increases intestinal perforation risk (1).

Gastrointestinal (GI) motility is essential for life and is a highly regulated and coordinated process. Research in GI motility started early in history from the observations of gastric contractions to the discovery of spontaneous colonic contractions in the cat tract using X-rays (2,3). Bayliss and Starling in 1899 (4) discovered that even after neural activity was blocked, myogenic contractions causing effective peristaltic activity occurred, providing evidence of an internal intestinal pacemaker. Cajal (5,6) proposed interstitial cells of Cajal (ICC) as an important player in GI motility via mediating enteric transmission, and later, Keith (7) proposed ICC as pacemakers.

The ICC generate spontaneously active pacemaker currents that may be recorded as plateau and slow potentials. These pacemaker currents drive the spontaneous electrical and mechanical activities of smooth muscle cells. The enteric nervous system (ENS), composed of both the myenteric (inter-muscular) plexus and the submucosal plexus, is also distributed in the gastrointestinal tract from the esophagus to the internal anal sphincter. We know that ICC contribute to several important functions in the GI tract including: 1) generation of electrical slow wave activity, 2) coordination of pacemaker activity and active propagation of slow waves, 3) transduction of motor neural inputs from the enteric nervous system, and 4) mechanosensation to stretch of GI muscles (8). Imaizumi et al. (9) believe that ICC mediate a component of the post-junctional response to enteric motor neurotransmission from morphological observations showing close contacts between varicosities of enteric nerves and ICC. This may play a role in transmitting stimuli received from the axon to surrounding smooth muscle cells by an electrotonic response. A morphometric study of the lower esophageal sphincter quantified close contacts between nerve varicosities and ICC *vs* contacts between varicosities and smooth muscle cells. ICC and nerve terminals were frequently in close contact, but similar contacts between nerve terminals and smooth muscle cells were more rare (10).

ICC form networks with a very broad distribution in the submucosal-ICC (ICC-SM), intra-muscular-ICC (ICC-IM), and inter-muscular layers (ICC-MY) of the GI tract. They generate spontaneously active pacemaker currents and this may drive self-generating and mechanical activity of smooth muscle cells. The ENS-ICC smooth muscle network is the main part of the regulation of GI motility (11–13). ICC form gap junctions with smooth muscle cells in visceral smooth muscles and provide important regulatory functions. They have an important role in mediating enteric neurotransmission. ICC are involved in transduction of neural impulses from peripheral nerves to bowel smooth muscle cells (7). In the small intestine, slow waves are initiated in the ICC network, which is located in the area where the myenteric plexus between circular and longitudinal muscle layers are also located (12). ICC-deep muscular plexus (ICC-DMP) is located between the inner and outer circular muscle sublayers in the small intestine (13). It forms a close synaptic contact with the nerve endings of the enteric motor neurons, and it is important for motor neuron reception. Kit signaling is essential for development and maintenance of ICC and electrical rhythmicity in the embryonic GI tract (14). Blocking of the c-Kit signaling pathway leads to the conversion of ICC to a smooth muscle-like phenotype. Little is known about the role of c-Kit's ligand-SCF (stem cell factor). After the interaction between *SCF* and its receptor c-Kit, the Kit signaling pathway can promote ICC development, proliferation, and function maintenance. This study investigated the *SCF* and c-Kit changes of ICC at different times in rat models of IO.

## Material and Methods

### Surgical procedure

All animal experiments of this study were approved by the Medical Ethics Committee of the Tianjin Institute of Integrated Traditional Chinese and Western Medicine Acute Abdominal Disease and animals were treated according to the guidelines of the National Institutes of Health for the care and use of laboratory animals. Male Wistar rats (n=40) aged between 40 and 60 days and with a body weight of 200–250 g were used for the study of IO. All efforts were made to minimize the number of rats used and their suffering. These animals were obtained from Chinese People's Liberation Army Academy of Military Medical Experimental Animal Center (China). They were randomly divided into 4 groups of 10 each: 1) group C: sham-operation control; 2) group M1: IO 1 day; 3) group M2: IO 2 days; and 4) group M3: IO 3 days.

**Figure 1.**
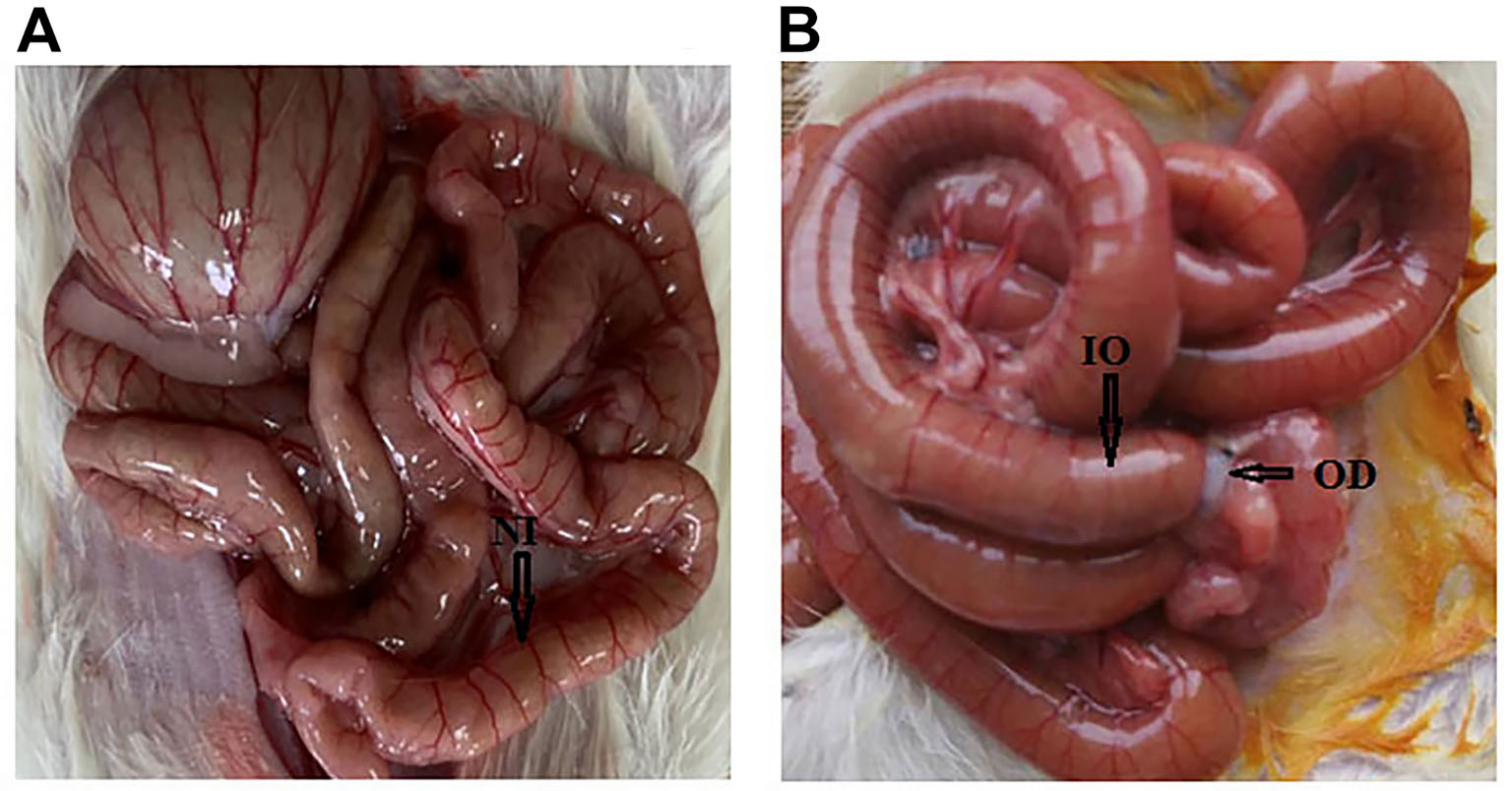
A, Normal intestinal tube (NT; arrow). **B**, Intestinal tube after 48 h of intestinal obstruction (IO; arrow) of experimental animals (short arrow indicates the obstruction device, OD).

All operations were performed using intraperitoneal injection of pentobarbital (40 mg/kg, Sinopharm Chemical Reagent Co., China). Surgery was performed to expose the loop of the intestine. In the animals with obstruction, a standard polyethylene ligating clip was placed across the terminal ileal lumen approximately 5 cm proximal to the ileocecal valve. After surgery, all rats were given standard rat food and water. Animals were killed by CO_2_ inhalation 24, 48, and 72 h after the initial surgery. An effective mechanical obstruction was established when the ileum above the ligation was greater than 50% of the ileum at the distal end of the ligation clip (Figure 1). Histologic examination of non-ischemic or ischemic ileum obstruction was performed for the evaluation of mucosal ischemic injury. Group C animals were prepared with the same surgical procedures but no clips were installed.

### Specimen collection

Immediately after death, the ileum was opened by an incision along the mesenteric border and laid flat. A 3-cm section of ileum tissue approximately 10 cm proximal to the ileocecal valve was obtained, the intestinal contents (yellow watery stool) were removed using Tyrode's solution, and the segment was washed 3 times with PBS and then dried with filter paper. Part of the specimen was placed in a 1.5-mL EP tube (stored at –80°C) and the remaining specimens were fixed in 10% neutral formalin buffer.

### Tissue RNA extraction and RT-PCR

RNA was extracted from all specimens using TRIzol. Briefly, 100 mg of homogenized tissue samples were mixed with 800 μL of TRIzol and 200 μL of chloroform. The mixture was vortexed thoroughly for 2 min, incubated for 20 min at room temperature, and centrifuged at 8,000 *g* for 20 min at 4°C. The upper phase was carefully transferred into a fresh tube, and precipitated with 600 μL isopropanol at –20°C for 20 h. After centrifugation, the RNA pellet was washed with 70% ethanol, air dried, and finally re-suspended in 1 mL diethyl pyrocarbonate (DEPC)-treated water. Five hundred nanograms RNA was converted to cDNA by reverse transcription according to TIANGEN (<http://www.tiangen.com/en/>, China) efficient reverse transcription kit instructions, and stored at –80°C.

Each real-time PCR reaction tube contained 12.5 μL SYBR Green Real-time PCR Master Mix, 1 μL of each forward and reverse primer (SCF forward: 5′-CATGCTTTAAGGCCTTTGTCACGAG-3′ and SCF reverse 5′-GGAGATGGCAGTTGTGCAGCTA-3′; GAPDH forward 5′-GGCACAGTCAAGGCTGAGAATG-3′ and GAPDH reverse 5′-ATGGTGGTGAAGACGCCAGTA-3′), 2 μL of cDNA template, and 6.5 μL dH_2_O. The mixture was centrifuged at 425 *g* at 4°C for 15 s, then real-time PCR was carried out using an initial denaturation step at 95°C for 10 min, followed by 40 cycles of amplification with denaturation at 95°C for 15 s, and annealing and extension at 60°C for 1 min. PCR signals from a total of 40 cycles were collected at 60°C and quantitative fluorescence was determined by real-time PCR software. The relative expression of target gene was calculated by the comparative CT method (2^-ΔΔ^CT method) using the following formulas: ^Δ^CT = CT target (SCF) – CT reference (GAPDH) and 2^-ΔΔ^CT = ^Δ^CT (IB groups) – ^Δ^CT (Control).

### Tissue protein extraction and western blotting

Total protein was extracted with RIPA lysis buffer (50 mM Tris-HCl pH 7.4, 150 mM NaCl, 0.1% Triton X-100, 1% PMSF) on ice. After centrifugation at 600 *g* at 4°C for 15 min, 20 µL of protein supernatants (100 μg/100 µL) were separated by sodium dodecyl sulfate-polyacrylamide gel electrophoresis (10%) and transferred to polyvinyl difluoride membranes (Millipore, USA). The membranes were blocked in TBST containing 0.1% Tween-20 (Solarbio Life Sciences, China) and 5% non-fat dry milk for 2 h at room temperature, and incubated with antibody to c-Kit (in 1:500 dilution SC-168, Santa Cruz Biotechnology, USA) and β-actin (in 1:2000 dilution, Cell Signaling Technology, USA) overnight at 4°C. Then, membranes were washed with TBST followed by 30 min incubation with horseradish peroxidase-conjugated secondary antibody (1:2000 dilution; Proteintech, China) at room temperature and detected using enhanced chemiluminescence (Thermofisher Scientific, USA). After color development, the image was analyzed using ChemiScope mini chemiluminescence meter to calculate the absorbance of the target and the internal control band for the protein expression: protein expression = integral absorbance value of target protein / integral absorbance value of β-actin.

### Immunohistochemistry for ICC positive cell counts

Intestinal samples were fixed with 4% paraformaldehyde and embedded in paraffin. After deparaffinization and dehydration, sections were boiled in Tris-EDTA buffer (10 mM Tris Base, 1 mM EDTA solution, 0.05% Tween 20, pH 9.0) for antigen unmasking, followed by extensive washing with PBS. Sections were subsequently incubated with 3% H_2_O_2_ for 10 min, and then rinsed with PBS three times. Sections were blocked with 2% goat serum albumin in PBS for 20 min and incubated with SCF antibodies (SC-168, in 1:150 dilution) for 2 h at 37°C. After 3 times washing with PBS, sections were incubated with anti-rabbit antibodies for 20 min at room temperature. Peroxidase activity was developed with diaminobenzidine (DAB) and the sections were counterstained with hematoxylin and dehydrated, hyalinized, and embedded. Finally, the specimen was mounted between a glass microscope slide and a glass coverslip and was visualized by a microscope (Olympus BX41, Olympus Corporation, Japan), with 400× magnification. The cells containing brown deposits of DAB (result of the immunohistochemical reaction) in the region of the muscular zone were considered positive and morphometrically assessed. ICCs were mainly distributed on the inner surface of the circular muscle, clinging to the smooth muscle cells, and running parallel to the smooth muscle (15). The SC-168 positive cells from 4 different fields of view from each animal (n=10 per group) were randomly selected to calculate the average number of ICC.

### Statistical analysis

Statistical analysis using SPSS 19.0 was performed for all data. The results are reported as means±SD. One-way ANOVA followed by Newman-Keuls test was used to identify significantly different means among the 4 groups. P<0.05 was considered statistically significant.

## Results

### Tissue *SCF* mRNA levels

Compared with group C, the relative expression levels of *SCF* mRNA in IO groups were significantly decreased at different time points. The degree of decline of *SCF* in M1 to M3 groups was increased with IO time (P<0.05, Table 1).

**Table 1.** Relative expression of *SCF* mRNA in rat intestinal tissue.

Groups	Fold change (2^-ΔΔ^ *C*T)
C	1.00±0.09*
M1	0.77±0.07*^#^
M2	0.37±0.06*^#^
M3	0.29±0.10*^#^

Data are reported as means±SD (n=10 per group). Intestinal tissues were from rats with sham-operation (C), one day obstruction (M1), two days obstruction (M2), and three days obstruction (M3). *P<0.05 among the 4 groups; #P<0.05 compared with Group C (one-way ANOVA followed by Newman-Keuls test).

### Tissue c-Kit protein expression levels

**Figure 2.**
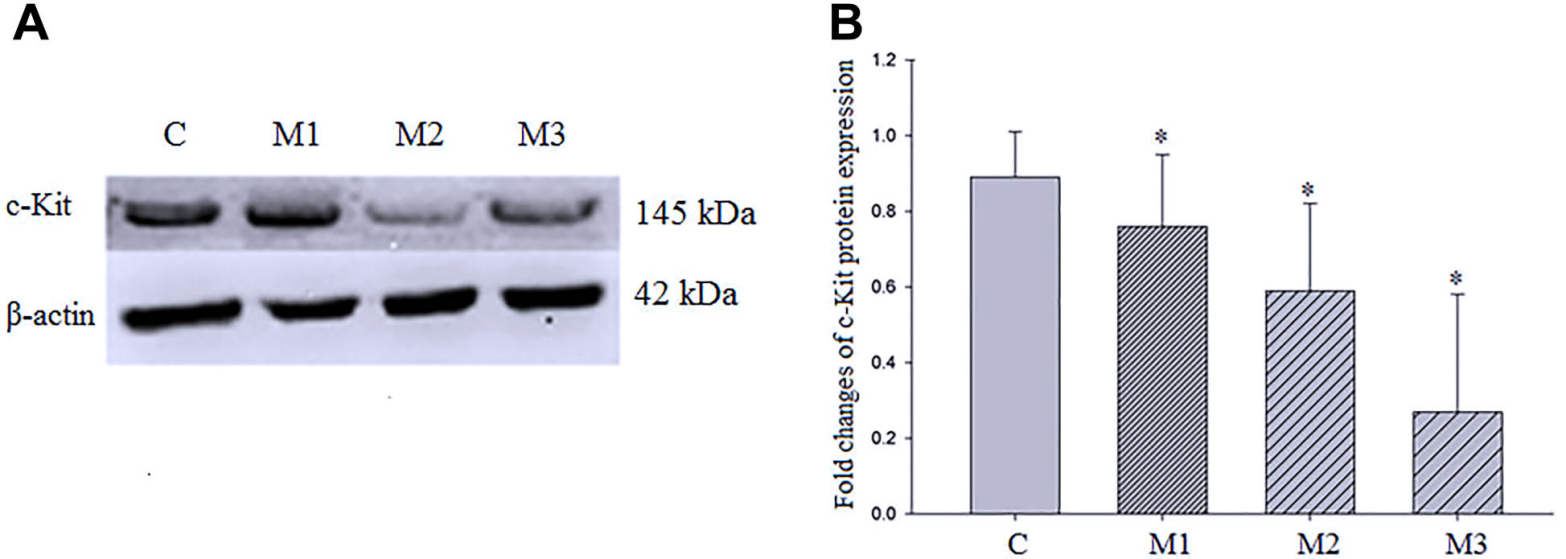
A, Western blotting detection of c-Kit protein expression of interstitial cells of Cajal after intestinal obstruction. Intestinal tissues were from rats with sham-operation (C), one day obstruction (M1), two days obstruction (M2), and three days obstruction (M3). **B**, Histogram of relative c-Kit protein expression. Results are reported as means±SD. *P<0.05 among the 4 groups (one-way ANOVA followed by Newman-Keuls test).

The relative gray values of groups C, M1, M2, and M3 were 0.89±0.12, 0.76±0.19, 0.59±0.23, and 0.27±0.31, respectively. Compared with group C, the expressions of c-Kit protein in M1 to M3 groups were significantly decreased. The degree of decline of c-Kit protein concentration in M1 to M3 groups was increased with IO time as well (P<0.05, Figure 2A and B).

### Immunohistochemistry of ICC

**Figure 3.**
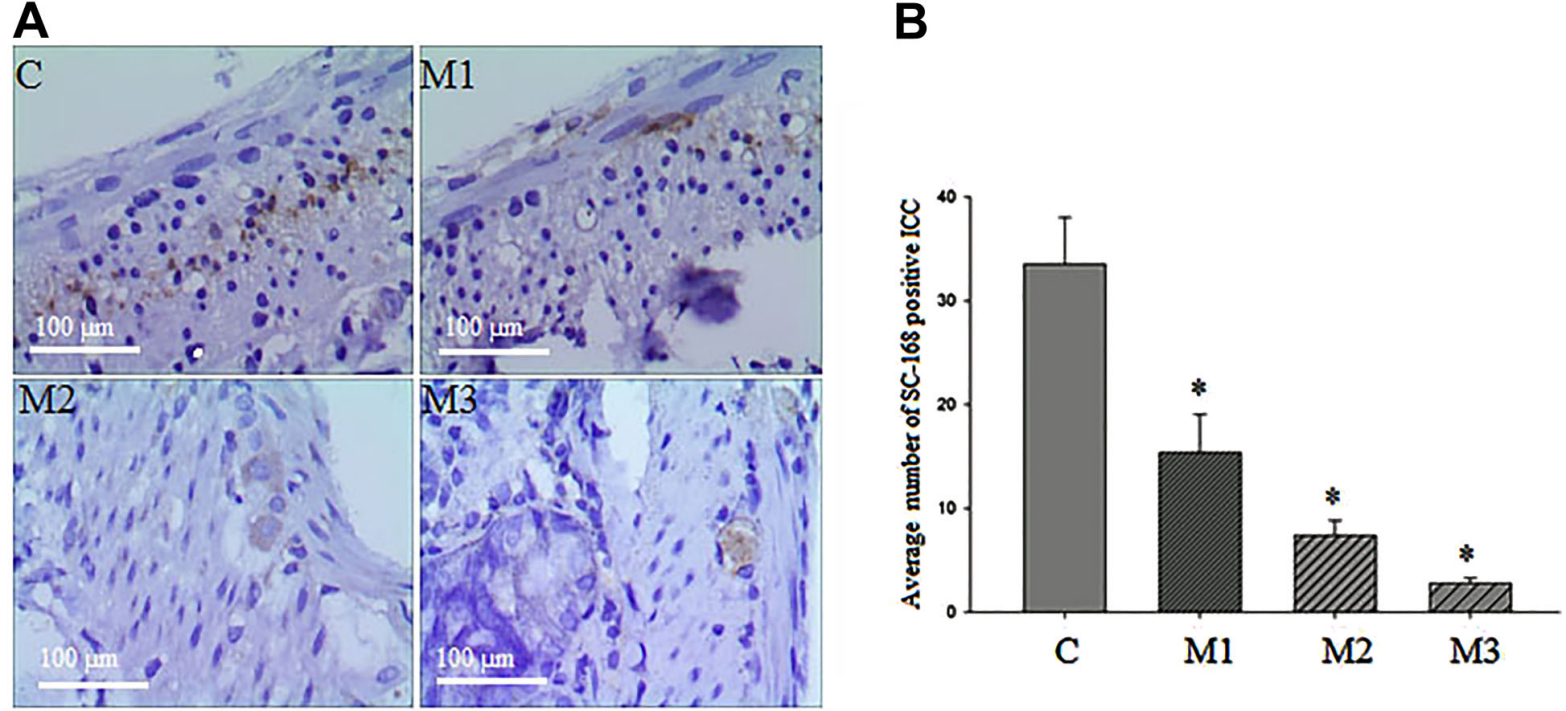
**A**, Immunohistochemical staining images of interstitial cells of Cajal (ICC) after intestinal obstruction (400× magnification; bar, 100 μm). Intestinal tissues are from rats with sham-operation (C), one day obstruction (M1), two days obstruction (M2), and three days obstruction (M3). **B**, Histogram of average number of ICC after immunohistochemical staining. Results are reported as means±SD. *P<0.05 among the 4 groups (one-way ANOVA followed by Newman-Keuls test).

Cells that showed brown staining were considered to be positive for ICC (Figure 3A). The number of ICC positive cells observed in group C was significantly increased compared to the experimental groups. With the prolongation of obstruction time, the number of ICC in experimental groups decreased, with a significant difference. The average number of ICC stained by SC-168 was 33.5±4.52 in C group, 15.4±3.69 in M1 group, 7.4±1.42 in M2 group, and 2.8±0.47 in M3 group (P<0.05, Figure 3B).

## Discussion

Among the biomarkers evaluated in acute intestinal ischemia, D-lactate, ischemia modified albumin (IMA), and intestinal fatty acid binding protein (I-FABP) are perhaps the most promising due to optimal sensitivity and relatively acceptable specificity (16). IO caused a significant decrease in *SCF* mRNA and c-Kit protein expression in ileum sections in a time-dependent manner. In the late stage of IO, the intestine is extremely dilated, the intestinal smooth muscle is damaged, and the intestine is congested and edematous, resulting in weakness of intestinal motility and imbalance of absorption (17).

ICC are mesenchyme-derived cells, belonging to the family of smooth muscle cells in which the activation of Kit signaling is required for their development. The discovery that ICC expresses c-Kit has helped to understand the morphology and physiological roles of ICC. Morphological studies have identified different phenotypic classes of ICC with different regulatory roles within the gut that help regulate the excitation-contraction coupling and the connectivity between smooth muscle cells and the motor output of the ENS (18).

ICC-MY cells lie within the myenteric plexus between the longitudinal and circular muscle layers and are regarded as the dominant pacemakers in gastric muscles. ICC-IM cells related to the DMP were seen within both the circular and longitudinal muscle layers. ICC-IM can also generate spontaneous depolarizations, referred to as unitary potentials or spontaneous transient (19–21). Relying on this potential, ICC-IM delivers pacing signals generated by ICC-MY to ICC-IM connected smooth muscle, thus completing the spontaneous contraction process.

One of the important breakthroughs in this field has been the discovery that the SCF/c-Kit signaling pathway is essential for normal development, maturation, and survival of ICCs, and is required for maintenance of phenotype and function of ICC networks. This finding was first suggested after observing that a severe anomaly of GI tract movement with depletion of ICCs resulted from the blockade of KIT postnatally by an antagonistic anti-KIT antibody. Numerous studies have subsequently revealed an association between loss of ICCs and disorder of the SCF/c-Kit signaling pathway in human clinical diseases (22,23).

The c-Kit receptor is the product of c-Kit proto-oncogene, belonging to the receptor tyrosine kinase (RTK) superfamily, which is the main regulator of mammalian cell fate (24). c-Kit is an important member of the type III RTK family, is highly specific, and has a restricted expression pattern, expressed in hematopoietic cells, mast cells, and on the surface of the ICC (25,26). The cognate ligand of c-Kit is SCF, which is synthesized in large quantities by smooth muscle cells of the GI tract (27). Therefore, the expression patterns of SCF and c-Kit are consistent with their potential participation in irritable bowel syndrome (28).

Our experiment in the small intestine showed that in IO rats the *SCF* expression decreased and the c-Kit protein content decreased. Immunochemical studies have shown a decrease in the number of ICC. With prolonged IO, the number of ICC gradually reduced, suggesting that regulation of SCF/c-Kit on ICC is closely related to the aberrant intestinal dynamics, including intestinal pacing and neurotransmission. These changes can cause GI motor dysfunction, manifested by altered intestinal motility and transit time, incoordination, or paralysis. The detection of SCF or c-Kit levels can indirectly indicate the number and functional status of ICC. The decrease of the level of SCF may be the reason the DMP induced intestinal/colonic structural and biomechanical remodeling.

ICC abnormality or decrease in number has been reported in some GI tract diseases. ICC have a dose-dependent and time-limited proliferation response to SCF (29). In addition, Tong et al. (30) revealed that exogenous SCF improved ICC number and function via the SCF/c-Kit pathway, with a high SCF concentration having increased potency. However, understanding the nature of the relationship between ICC and the generation of human gut motility disorders and the underlying mechanism for ICC loss is still in progress (17). There are two types of SCF: soluble SCF (sSCF) and membrane-bound SCF (mSCF). sSCF mainly maintains the number of ICC precursor cells (31), while mSCF can differentiate ICC precursor cells into mature ICC network (32,33). Therefore, the decrease of SCF expression can cause ICC number and ultrastructural abnormalities (34).

Our results showed that SCF/c-Kit signaling is crucial for the development of ICC biological function. At the cellular level, the expression trend of *SCF* gene and c-Kit protein is basically the same. Increasing the number of ICC or changing the expression of c-Kit protein, which is its marker, is conducive to the recovery of the ICC network structure and promotes the recovery of intestinal function. In this experiment, ICC phenotypic transduction, as well as ICC number reduction and network destruction caused by mechanical IO in rats, provided a good experimental model for further study on the cellular/molecular mechanism of ICC phenotypic changes.

### Conclusions

IO caused a reduction in the concentration of *SCF* mRNA and c-Kit proteins in ICC. With prolonged IO, the number of ICCs decreased. The expression levels of *SCF* mRNA and c-Kit protein in ileum sections were significantly reduced with increasing IO time.
